# Targeting 7KCh-Induced Cell Death Response Mediated by p38, P2X7 and GSDME in Retinal Pigment Epithelium Cells with Sterculic Acid

**DOI:** 10.3390/pharmaceutics15112590

**Published:** 2023-11-05

**Authors:** Ana Pariente, Rafael Peláez, Rodrigo Ochoa, Álvaro Pérez-Sala, Ángela Villanueva-Martínez, Miriam Bobadilla, Ignacio M. Larráyoz

**Affiliations:** 1 Biomarkers and Molecular Signaling Group, Neurodegeneration Area, Center for Biomedical Research of La Rioja (CIBIR), Piqueras 98, 26006 Logroño, Spain; apariente@riojasalud.es (A.P.); rpelaez@riojasalud.es (R.P.); rochoa.iacs@aragon.es (R.O.); aperez@riojasalud.es (Á.P.-S.); avmartinez@riojasalud.es (Á.V.-M.); 2 Proteomics Research Core Facility, Aragonese Institute of Health Sciences (IACS), San Juan Bosco 13, 50009 Zaragoza, Spain; 3 Biomarkers, Artificial Intelligence and Signaling (BIAS), Department of Nursing, University of La Rioja, Duquesa de la Victoria 88, 26006 Logroño, Spain

**Keywords:** 7-ketocholesterol, sterculic acid, AMD, retina, cell death, P2X7, GSDME

## Abstract

Age-related macular degeneration (AMD) is the main cause of blindness in developed countries. AMD is characterized by the formation of drusen, which are lipidic deposits, between retinal pigment epithelium (RPE) and the choroid. One of the main molecules accumulated in drusen is 7-Ketocholesterol (7KCh), an oxidized-cholesterol derivative. It is known that 7KCh induces inflammatory and cytotoxic responses in different cell types and the study of its mechanism of action is interesting in order to understand the development of AMD. Sterculic acid (SA) counteracts 7KCh response in RPE cells and could represent an alternative to improve currently used AMD treatments, which are not efficient enough. In the present study, we determine that 7KCh induces a complex cell death signaling characterized by the activation of necrosis and an alternative pyroptosis mediated by P2X7, p38 and GSDME, a new mechanism not yet related to the response to 7KCh until now. On the other hand, SA treatment can successfully attenuate the activation of both necrosis and pyroptosis, highlighting its therapeutic potential for the treatment of AMD.

## 1. Introduction

Age-related macular degeneration (AMD) is the leading cause of legal blindness in people older than 65 years in developed countries. This disease affects the macula, is related to aging and is characterized by a gradual loss of central vision [1,2,3,4]. There are mainly two types of AMD, called dry AMD and wet AMD, of which dry AMD is the milder and more common form of the disease [1,5,6]. As a consequence of aging, waste material not digested by retinal pigment epithelium cells (RPE) accumulates around it, forming yellowish specific extracellular deposits called drusen, which is the first hallmark of the disease [6,7,8,9,10]. Given the importance of the RPE in drusen formation, currently, much of the research focuses on this part of the retina [11]. The increase in the number and size of drusen causes the molecules inside them, mainly lipid compounds, cholesterol derivatives and unfolded-oxidized proteins, to start promoting different inflammatory and cytotoxic responses that damage the RPE, triggering the development of dry AMD [3,8,10,12,13]. Due to its slow progress, dry AMD does not usually provoke the complete loss of vision [3].

Wet AMD, by contrast, is the most severe form of AMD and it is characterized by the abnormal growth of choroidal vessels from the macula through the RPE in a process called choroidal neovascularization (CNV) [1,3,6,11]. At some point, during this process, blood and fluids will leak, damaging the macula and causing the irreversible loss of central vision [5,6]. Anti-VEGF therapies are the only treatment currently available for AMD patients, which are not exempt from serious side effects. In addition, there are patients who do not respond well or can develop resistance, causing a decrease in the efficacy of these treatments [1,5,6,14,15,16]. For these reasons, there is an urgent need to search for new therapeutic targets that allow the development of new therapies.

One of these alternative therapeutic targets is 7-Ketocholesterol (7KCh), an oxidized form of cholesterol or oxysterol strongly connected with the development of AMD, as it forms and accumulates in drusen as a consequence of aging [17,18,19,20,21,22]. It is known that 7KCh can trigger oxidative stress, inflammation and cell death responses in different cell types, including retinal cells, although its response seems to depend on the cell type and the dose used [13,23,24,25,26,27,28,29,30,31,32,33,34]. The investigation of the mechanism of action of 7KCh in retinal cells could provide a new perspective on the molecular mechanisms that can lead to the development of AMD.

**Figure 1:**
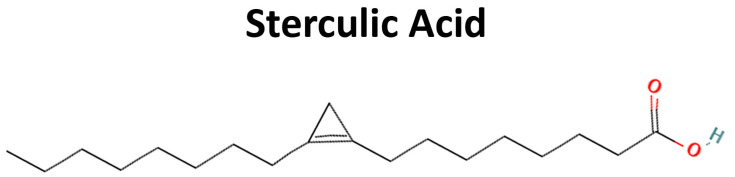
Chemical structure of sterculic acid (image obtained from Pubchem, https://pubchem.ncbi.nlm.nih.gov/compound/Sterculic-acid#section=2D-Structure, accessed on 26 October 2023, CID: 12921).

On the other hand, sterculic acid (SA), a natural cyclopropane fatty (Figure 1) acid obtained from the plant *Sterculia foetida*, has been described as the most potent functional antagonist of 7KCh in retinal cells [23,24,33,34,35], being able to protect RPE cells against 7KCh deleterious effects at low concentrations, as well as being able to reduce laser-induced choroidal neovascularization in a rat model [23,24,34]. These results suggest that SA could be a good alternative to the treatment of ocular diseases, such as AMD [35,36]. SA is known to inhibit Stearoyl coenzyme-A desaturase 1 (SCD1) activity, an enzyme from de novo lipogenesis, both in vitro and in vivo [37,38,39]. However, its protective effect against the 7KCh response is independent of the inhibition of SCD1 [35,36].

In our previous work, we performed genome-wide transcriptomic analysis in RPE cells in order to unravel the molecular pathways induced by 7KCh in these cells, as well as the mechanisms by which SA is able to protect against this response. We found that 7KCh exposure modulates the expression of several genes associated with lipid metabolism, reticulum stress, inflammation and cell death and activates signaling pathways related to these genes, such as unfolded protein response (UPR), the classical MAPK pathway and the JNK and p38 pathways. Co-treatment with SA significantly reversed gene expression alterations as well as attenuated the activation of these pathways [34]. In the present work, we continued with the study of the signaling induced by 7KCh in RPE cells, focusing on the cell death pathways activated by this oxysterol. Similarly, we have evaluated the protective effect of SA on the 7KCh-induced cytotoxic response.

## 2. Materials and Methods

### 2.1. Cell Lines and Culture

mRPE cells were obtained as a gift from Dr. SP Becerra, from the National Eye Institute (NIH, Bethesda, MD, USA). These cells came from the eyes of 3- to 5-year-old Rhesus monkeys, and were maintained for two weeks in a monolayer until biochemical and physiological markers characteristic of differentiated tissue were expressed [40]. DMEM/F12 1:1 medium (Hyclone-Thermo Scientific, Waltham, MA, USA) was used to grow the cells. Cell culture medium was supplemented with 5% fetal bovine serum (Invitrogen, Alcobendas, Madrid, Spain), 1.5% of sodium pyruvate, 1% of penicillin/streptomycin and 1% of non-essential amino-acids (Hyclone-Thermo Scientific, Waltham, MA, USA). Cultured cells were kept in an atmosphere of 37 °C, 5% CO_2_ and 85% humidity.

### 2.2. Cell Treatments

Cells were seeded in P100 plates for DNA, RNA and protein purification at a density of 1.2 × 10^6^ cells/plate or in 12-well plates for cell viability assays at a density of 100,000 cells/plate. Cells were allowed to attach and 100% confluency was reached after 24 h after seeding. Then, the culture media was replaced with serum-free media for 24 h and, after that, treatments were added. Cells were treated with 15–20 μM 7KCh (Sigma-Aldrich, Madrid, Spain) alone or with 10 μM SA (chemically synthesized by the Center for Applied Chemistry and Biotechnology, PPQF, University of Alcalá, Alcalá de Henares, Spain), 1–10 μg/mL og Cycloheximide (CHX, Sigma-Aldrich, Madrid, Spain), 5–25 μM of Caspase 3 inhibitor Z-DEVD-FMK (Merck, Darmstadt, Germany), 3–10 μM of RIPK1 inhibitor Necrostatin-1 (Nec-1, Cayman Chemical, Ann Arbor, MI, USA), 0.5–2 μM of MLKL inhibitor Necrosulfonamide (NSA, Tocris, Bristol, UK), 5 μM of P2X7 inhibitor A-839977 (Santa Cruz Biotechnology, Dallas, TX, USA), 25–75 μM of pannexin-1 inhibitor Carbenoxolone (CBX, Sigma-Aldrich, Madrid, Spain), 20 μM of JNK inhibitor SP600125 (StressMarq Bioscience Inc., Victoria, BC, Canada) or 40 μM of p38 inhibitor SB203580 (Sigma-Aldrich, Madrid, Spain). Then, 7KCh was prepared in β-cyclodextrin (Sigma-Aldrich, Madrid, Spain) as previously described [13,24]; SA, Z-DEVD-FMK, Nec-1, NSA, A-839977, CBX, SP600125 and SB203580 were dissolved in DMSO (Sigma-Aldrich, Madrid, Spain) and CHX was dissolved in absolute ethanol.

### 2.3. Cell Viability Assays

CellTitter 96 Aqueous One Solution Reagent MTS assay (Promega, Madison, WI, USA) was used to validate the toxicity or protective effect of the different treatments following the manufacturer’s guidelines. Cells were washed two times with 1X PBS and then MTS reagent was added at a ratio of 1:10 in culture medium. Plates were incubated at 37 °C and absorbance was measured at baseline and after 4 h of incubation using a Biotek Synergy H4 multi-mode plate reader (BioTek Instruments, Covina, CA, USA). The results were presented as a percentage of viability with respect to the control cells.

### 2.4. DNA Laddering

Cell extracts were obtained from cells in culture, collecting both floating cells and cells adhered to the plate. Once harvested, cells were centrifuged (1000 rpm, 5 min) to remove residual media and washed with 1× PBS at 4 °C. The resulting pellet was resuspended in 250 μL of Tris-EDTA (tris (hydroxymethyl) aminomethane-ethylenediaminetetraacetic acid)/Triton X-100 buffer (10 mM Tris pH 8, 1 mM EDTA and 0.2% *v*/*v* Triton X-100, Sigma-Aldrich, Madrid, Spain) to extract the DNA from the cells by lysis. Then, extracts were incubated for 10 min on ice and centrifuged for 15 min at 4 °C and 13,000× *g*. The supernatant was then incubated with 10 mg/mL RNAse-A (Macherey-Nagel, Dueren, Germany) at 37 °C for 1 h, and with 20 μM proteinase K (Abm, Richmond, BC, Canada) and 10% SDS (sodium dodecyl sulfate, Sigma-Aldrich, Madrid, Spain) at 50 °C for 1 h in order to remove RNA and protein debris. To precipitate the DNA, 0.1 volume of 5 M NaCl (Sigma-Aldrich, Madrid, Spain) and one volume of isopropanol (Sigma-Aldrich, Madrid, Spain) at −20 °C were added to the samples, which were incubated on ice for 10 min. Afterward, the samples were centrifuged for 15 min at 4 °C and 13,000× *g*, and the resulting pellet was resuspended in 12 μL of Tris-EDTA buffer (10 mM Tris pH 8, 1 mM EDTA). Finally, the DNA concentration was calculated using a NanoDrop 1000 spectrophotometer (Thermo Fisher Scientific, Madrid, Spain) using Tris-EDTA buffer as a blank.

The DNA laddering pattern of bands was visualized by running 1 μg of DNA from each sample mixed with loading buffer (6× Loading buffer, Thermo Fisher Scientific, Madrid, Spain) on 2% agarose gel electrophoresis in TAE buffer (Tris-Acetate-EDTA, Alfa Aesar, Haverhill, MA, USA) with GelRed^®^ (10,000× GelRed Nucleid Acid Stain, Biotium, Fremont, CA, USA) as the nucleic intercalating agent. Gels were run for 3 h at 50 V and bands were visualized under UV light in a Gel-Doc EQ System Universal Hood II transilluminator (Bio-Rad, Hercules, CA, USA). Images were taken using the Quantity One 4.6.3. program (Bio-Rad, Hercules, CA, USA).

### 2.5. In Vivo Immunofluorescence

DNA fluorescent staining was used to study the morphology of the cell nucleus in order to identify the different types of cell death depending on the state of the DNA. Cells were stained in vivo with 1:2000 Hoechst 33342 (Sigma-Aldrich, Madrid, Spain), 1:2000 propidium iodide (Sigma-Aldrich, Alcobendas, Madrid, Spain) or 1:10,000 YO-PRO1 (Invitrogen, Alcobendas, Madrid, Spain). The fluorochromes were directly added to the medium, to avoid losing dead cells floating in the medium, and incubated for 20 min at 37 °C. Cells were visualized and photographed at various times using a TSC SP5 confocal microscope (Leica, Wetzlar, Germany) in the case of Hoechst and propidium iodide co-staining, or the Incucyte^®^ Live-Cell Analysis System (Sartorius, Göttingen, Germany) in the case of propidium iodide and YO-PRO1 co-staining. Image analysis was carried out using the Image J 1.52h (Fiji, National Institute of Health, Bethesda, MD, USA) and Incucyte^®^ 2022A (Sartorius, Göttingen, Germany) programs, respectively. The excitation/emission wavelengths were 350/461 nm for Hoechst, 535/625 nm for propidium iodide and 488/515 nm for YO-PRO1.

### 2.6. RNA Purification and Quantitative Real-Time PCR (qRT-PCR)

Total RNA was isolated from cell cultures in 1 mL of TRIzol (Invitrogen, Madrid, Spain) and purified using the RNAsy mini kit (Qiagen, Valencia, CA, USA) and DNAse I (Qiagen, Valencia, CA, USA) following the manufacturer’s instructions. Then, reverse-transcription was carried out with 1 μg of total RNA, random primers and the SuperScript III kit (Invitrogen, Madrid, Spain) to obtain the first-strand cDNA. Then, cDNA was mixed with forward and reverse primers to a concentration of 0.3 μM (Table 1) and SYBR Green PCR Master Mix. Then, a 7300 Real-Time PCR System (Applied Biosystems, Madrid, Spain) was used to take quantitative measurements in the following cycling conditions: initial denaturation at 95 °C for 10 min, followed by 40 cycles of 95 °C for 15 s and 60 °C for 1 min. In order to validate the amplicon specificity, a dissociation curve was implemented from 60 °C to 95 °C. The interpolation of the Ct value from the corresponding standard line was used to calculate the gene expression levels using *18S* rRNA expression to normalize the results.

### 2.7. Western Blotting

Protein extracts were obtained using RIPA buffer (Thermo Scientific, Madrid, Spain) containing protease (EDTA-free complete, Roche, Basilea, Switzerland) and phosphatase (PhosStop, Roche, Basilea, Switzerland) inhibitors from scraped cells in culture. The centrifugation of homogenates for 30 min at 15,000× *g* was performed to collect supernatants and the protein concentration was determined using a BCA kit (Pierce, Rockford, IL, USA) and a Biotek Synergy H4 multi-mode plate reader (BioTek Instruments, Covina, CA, USA), following the manufacturer’s instructions. Samples were prepared by mixing 20 μg of protein with 4× sample buffer (Invitrogen, Madrid, Spain) and 10× sample-reducing agent (Invitrogen, Madrid, Spain), and were then heated at 70 °C for 10 min. Then, 4–12% SDS-polyacrylamide gels (Invitrogen, Madrid, Spain) were used to run both the samples and the molecular weight marker SeeBlue plus 2 prestained standard (Invitrogen, Madrid, Spain). Then, protein transfer was performed onto 0.2 μm polyvinylidene difluoride (PVDF) membranes (iBlot system, Invitrogen, Madrid, Spain) and blocking was carried out at room temperature for 2 h with 5% non-fat dry milk in 1× TBS (tris buffered saline) and 0.1% Tween (Sigma-Aldrich, Madrid, Spain).

For protein identification, membranes were incubated overnight at 4 °C with different primary antibodies: α-p-p38 1:5000 (#4511, Cell Signaling, Danvers, MA, USA), α-p38 1:5000 (#8690, Cell Signaling, Danvers, MA, USA), α-GSDMD 1:1000 (ab210070, Abcam, Cambridge, UK), α-GSDME 1:1000 (ab215191, Abcam, Cambridge, UK) or α-cleaved-Caspase 3 1:1000 (#9664, Cell Signaling, Danvers, MA, USA). The detection of α-Actin 1:10,000 (#A5441, Sigma-Aldrich, Madrid, Spain) was utilized to standardize protein levels. To visualize immunoreactivity, membranes were incubated with α-rabbit (#7074, Cell Signaling, Danvers, MA, USA) or α-mouse (715-035-1514, Jackson Immunoresearch Lab. West Grobe, PA, USA) and chemiluminescence Clarity Western ECL kit (Bio-Rad, Hercules, CA, USA), and exposed to X-ray films (Amersham Hyperfilm ECL, GE Healthcare, Buckinghamshire, UK) or the ChemiDoc Imaging System (Bio-Rad, Hercules, CA, USA).

### 2.8. Statistical Analysis

GraphPad Prism 6 was used to perform statistical analysis. Data were presented as means ± SEM (standard error of the means) and were considered statically significant when *p* ≤ 0.05. Normally distributed data were analyzed by ANOVA testing followed by Sidak or Tukey’s post hoc test, following program recommendations. Student’s *t*-test was used for comparisons of two conditions.

## 3. Results

### 3.1. 7KCh-Induced Cell Death Is Not Mediated by Apoptosis in mRPE Cells

**Figure 2:**
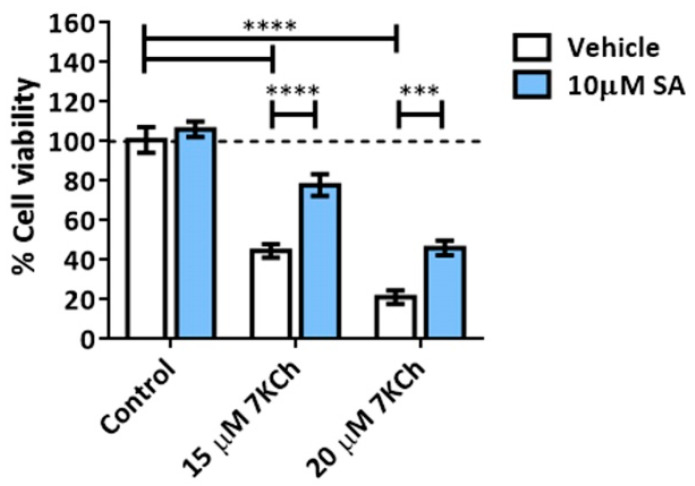
Cell toxicity induced by 7KCh and protective effect exerted by SA in mRPE cells. Cell viability was determined by MTS assay in mRPE cells treated with 15–20 μM 7KCh and 10 μM SA for 24 h. Data are expressed over the control and presented as a mean ± SEM of at least three different experiments. The dashed line is a guidance mark of the control value. The ANOVA test was used for statistical analysis, followed by the Sidak post hoc test. *** *p* < 0.001; **** *p* < 0.0001.

**Figure 3:**
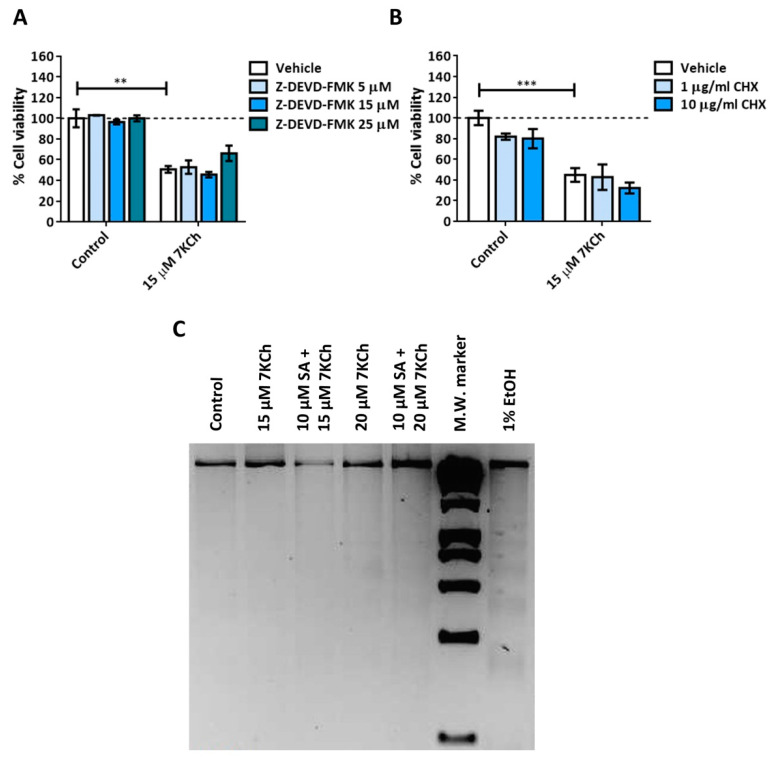
Participation of apoptosis in the toxic response induced by 7KCh in retinal cells. (**A**) Cell viability was determined by MTS assay in mRPE cells treated with 15 μM 7KCh and 5–25 μM Z-DEVD-FMK for 24 h. (**B**) Cell viability was determined by MTS assay in mRPE cells treated with 15 μM 7KCh and 1–10 μg/mL CHX for 24 h. (**C**) DNA laddering was detected by electrophoresis in mRPE cells treated with 15–20 μM 7KCh and 10 μM SA for 24 h. CHX and Z-DEVD-FMK were added with pretreatment for 2 h with respect to 7KCh. M.W., molecular weight. Data are expressed over the control and represented as mean ± SEM of at least three different experiments. The dashed line is a guidance mark of the control value. The ANOVA test was used for statistical analysis, followed by the Sidak post hoc test. ** *p* < 0.01; *** *p* < 0.001.

We treated mRPE cells with 15–20 μM 7KCh with or without 10 μM SA for 24 h (Figure 2) in order to validate the toxicity effect of 7KCh and the protection of SA, as previously described [34,35]. Once again, a dose-dependent decrease in cell viability was observed with 7KCh treatment, as well as a decrease in this effect when adding SA. With the aim to find out the type of cell death induced by 7KCh in mRPE cells, we evaluated the participation of apoptosis in this cytotoxic response, as in our previous work, we showed an alteration in the expression of several genes involved in this pathway, such as *APAF1*, *GADD45A*, *PMAIP1* or *TP53I3* [34]. First, cells were treated with the Caspase 3 inhibitor Z-DEVD-FMK in the concentration range of 5–25 μM and 15 μM 7KCh (Figure 3A). No protective effect was observed over 7KCh-induced cell death. Since apoptosis involves the synthesis of specific proteins to be activated [41,42], such as PUMA or NOXA, the next approach was to inhibit protein synthesis with CHX. Treatment with 1–10 μg/mL CHX for 24 h could not protect mRPE cells against the toxicity of 15 μM 7KCh (Figure 3B). Lastly, we evaluated whether treatment with 15–20 μM 7KCh with or without 10 μM SA caused DNA laddering fragmentation, characteristic of apoptosis, in mRPE cells. However, the ladder fragmentation pattern observed in the positive control (1% *v*/*v* ethanol) did not occur in 7KCh-treated cells (Figure 3C), indicating that apoptosis is not induced in response to 7KCh in mRPE cells.

### 3.2. Activation of Necrosis in Response to 7KCh in mRPE Cells

**Figure 4:**
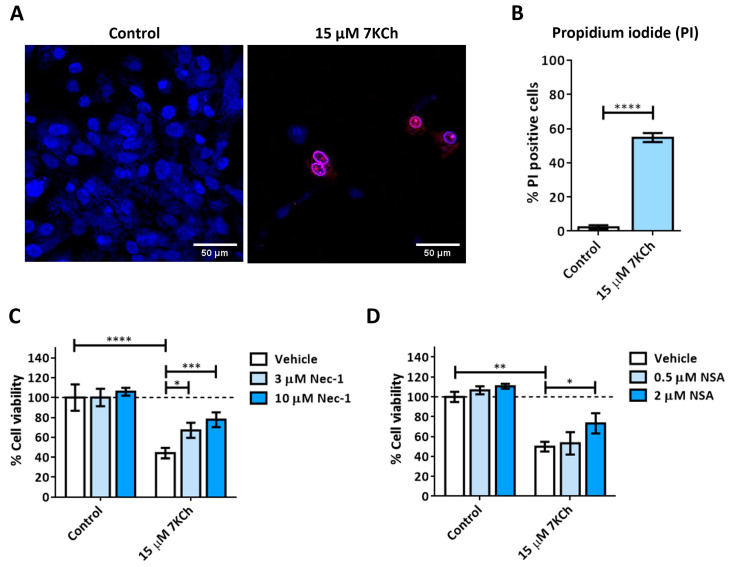
7KCh-induced necrosis in mRPE cells. (**A**) Representative images obtained in vivo by confocal microscopy in the control and 7KCh-exposed mRPE cells for 24 h stained with Hoechst (blue) and propidium iodide (IP, red). White arrow indicates an example of necrotic core. (**B**) Quantification of the percentage of nuclei stained with propidium iodide. Data expressed as the mean ± SEM of seven independent fields. (**C**) Cell viability determined by MTS assay in mRPE cells trated with 15 μM 7KCh and 3–10 μM Nec-1 or (**D**) 0.5–2 μM NSA. Data are expressed over the control and represented as mean ± SEM of at least three different experiments. Dashed line is a guidance mark of the control value. The ANOVA test was used for statistical analysis, followed by the Sidak post hoc test. * *p* < 0.05; ** *p* < 0.01; *** *p* < 0.001; **** *p* < 0.0001.

We then studied the role of necrosis in the toxicity mechanisms of 7KCh in mRPE cells. The control and 15 μM 7KCh-treated cells were stained with Hoechst and propidium iodide to assess for the presence of necrotic nuclei (Figure 4A). The percentage of mRPE cells exposed to 7KCh positive for propidium iodide was approximately 55% with respect to the total number of cells observed quantified with Hoechst staining (Figure 4B). In addition, the nucleus of these cells presented a ring shape (white arrows), with the genetic material being released from the nucleus (Figure 4A), as occurs in necrosis. In order to verify the implication of necrosis processes in the 7KCh-induced response, mRPE cells were also treated with two necroptosis inhibitors: 3–10 μM Necrostatin-1 (Nec-1) as the RIPK1 inhibitor (Figure 4C), and 0.5–2 μM Necrosulfonamide (NSA) as the MLKL inhibitor (Figure 4D). Both inhibitors could successfully attenuate 15 μM 7KCh-induced toxicity. Taken together, these results indicate that at least a significant part of the 7KCh-induced cell death in mRPE cells is mediated by necrotic processes.

### 3.3. The P2X7 Receptor Is Involved Is the Cell Death Response to 7KCh in mRPE Cells and Participates in p38 Activation and in Necrotic Processes

**Figure 5:**
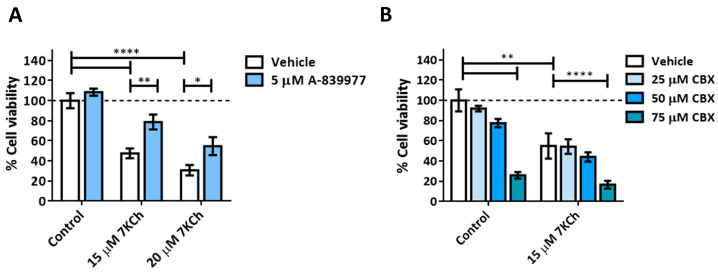
Effect of P2X7 inhibition on 7KCh-induced cell death. (**A**) Cell viability was determined by MTS assay in mRPE cells treated with 15–20 μM 7KCh and 5 μM A-839977 for 24 h. (**B**) Cell viability was determined by MTS assay in mRPE cells treated with 15 μM 7KCh and 25–75 μM CBX. Data are expressed over the control and represented as mean ± SEM of at least three different experiments. The dashed line is a guidance mark of the control value. The ANOVA test was used for statistical analysis, followed by the Sidak post hoc test. * *p* < 0.05; ** *p* < 0.01; **** *p* < 0.0001.

A pannexin-1-independent involvement of the P2X7 purinoceptor in the 7KCh-initiated necrosis response has been previously described in ARPE-19 cells [22], so we decided to check whether the same occurs in the mRPE cell line. Cells were treated for 24 h with the P2X7 inhibitor A-839977 at a concentration of 5 μM or with the pannexin-1 inhibitor Carbenoxolone (CBX) at concentrations of 25–75 μM in combination with 15–20 μM 7KCh, and cell viability was evaluated with MTS assay. The addition of A-839977 (Figure 5A), but not CBX (Figure 5B), protected mRPE cells against 7KCh cell death, indicating the participation of P2X7 in the cytotoxic response induced by 7KCh, but with the independence of pannexin 1.

**Figure 6:**
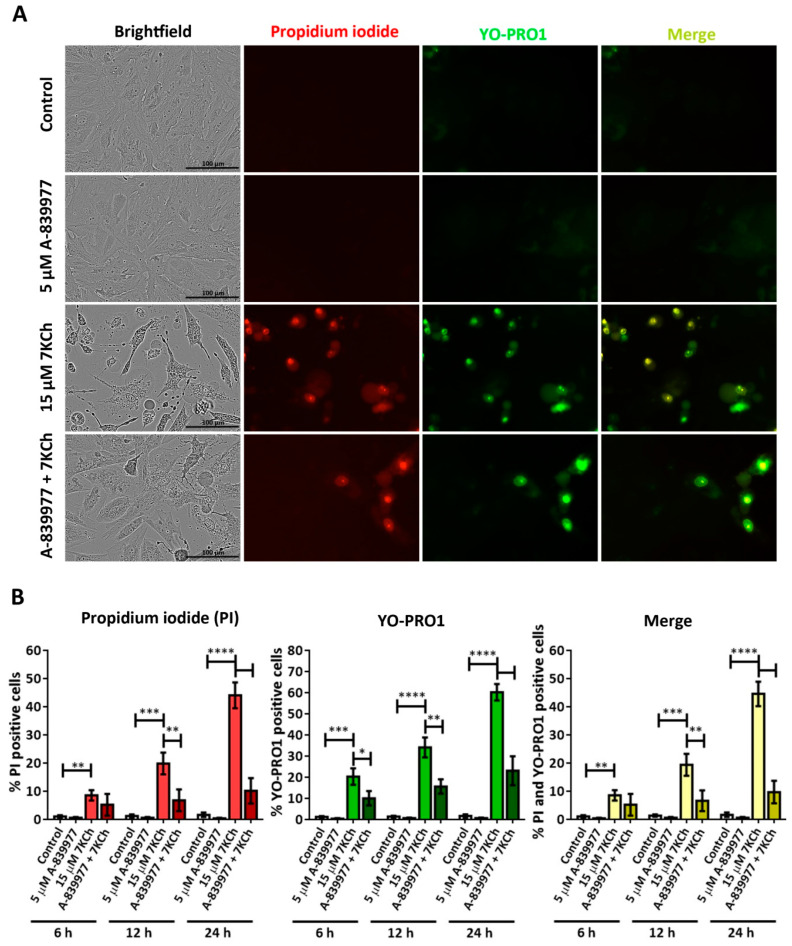
Involvement of the P2X7 receptor in 7KCh-induced toxicity in mRPE cells stained with propidium iodide (red) and YO-PRO 1 (green). (**A**) Representative images obtained in vivo in brightfield and fluorescence of mRPE cells treated with 15 μM 7KCh and/or 5 μM A-839977 for 24 h using Incucyte^®^ equipment. Merge (yellow) represents the combination of propidium iodide and YO-PRO1 fluorescence. (**B**) Percentage of cells stained with propidium iodide (IP), YO-PRO1 and both fluorochromes (merge) at 6 h, 12 h and 24 h of treatment with respect to the total cell number quantified in brightfield. Data are expressed as the mean ± SEM of four independent fields. The ANOVA test was used for statistical analysis, followed by Tukey’s post hoc test. * *p* < 0.05; ** *p* < 0.01; *** *p* < 0.001; **** *p* < 0.0001.

To further study the role of P2X7 in cell death induced by 7KCh, YO-PRO1 was used to label the cells, as this fluorochrome is capable of entering through the pores formed by this receptor when activated [43]. Cells were treated with 5 μM A-839977 and 15 μM 7KCh, and then simultaneously stained with propidium iodide and YO-PRO1 (Figure 6A). Cells positive for propidium iodide (red), YO-PRO1 (green) or both fluorochromes (merge) were quantified with respect to the total number of cells observed in the brightfield at 6 h, 12 h and 24 h. In mRPE cells treated with 7KCh, an increase in the number of nuclei positive for propidium iodide and/or YO-PRO1 was observed (Figure 6B). Moreover, the internalization of YO-PRO1 occurred before the internalization of propidium iodide since, at all times, the evaluated percentage of cells stained with YO-PRO1 was higher than that of cells stained with propidium iodide, although the difference narrowed over time. Approximately 8% of cells stained with propidium iodide and 20% with YO-PRO1 were observed at 6 h; 20% of cells were stained with propidium iodide and 33% with YO-PRO1 at 12 h; and 45% of cells were positive for propidium iodide and 60% for YO-PRO1 at 24 h. In addition, practically all cells positive for propidium iodide were also positive for YO-PRO1 (Figure 6B, merge). P2X7 inhibition with A-839977 significantly reduced the number of cells stained with both propidium iodide and YO-PRO1 at 12 h and 24 h (also at 6 h in the case of YO-PRO1). This indicates that in response to 7KCh, the P2X7 receptor is activated before necrosis begins and, in turn, participates in its induction. Furthermore, the percentage of cells solely stained with YO-PRO1 suggests the involvement of at least a second type of cell death, in addition to necrosis, in response to 7KCh-induced toxicity in mRPE cells.

### 3.4. SA Prevents the Activation of Necrosis and P2X7 Induced by 7KCh in mRPE Cells

**Figure 7:**
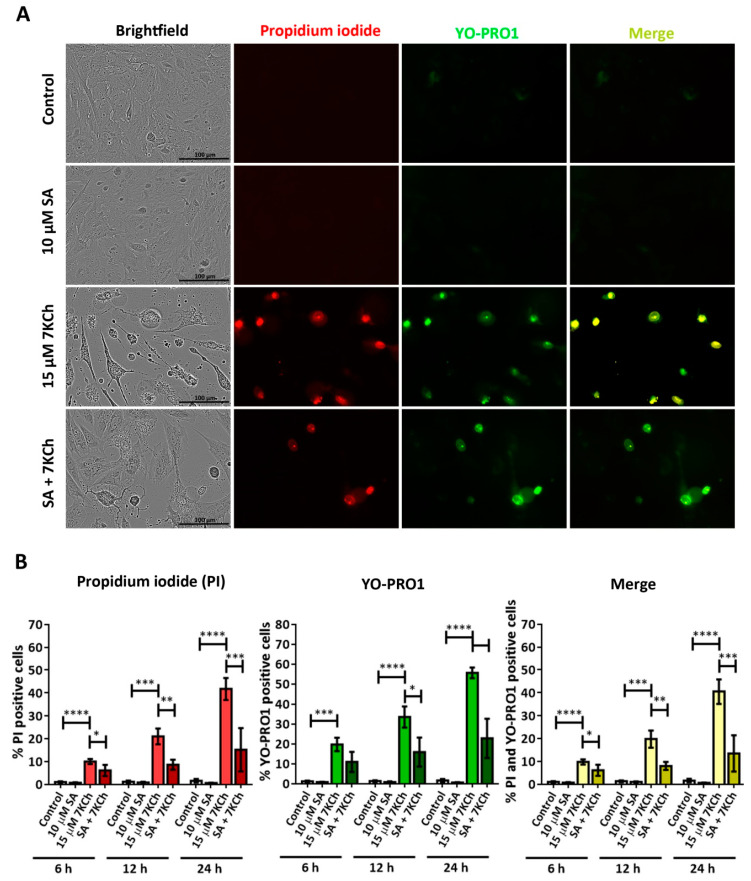
SA attenuation of necrosis and P2X7 activation induced in response to 7KCh in mRPE cells stained with propidium iodide (red) and YO-PRO1 (green). (**A**) Representative images obtained in vivo in brightfield and fluorescence of mRPE cells treated with 15 μM 7KCh and/or 10 μM SA for 24 h using Incucyte^®^ equipment. Merge (yellow) represents the combination of propidium iodide and YO-PRO1 fluorescence. (**B**) Percentage of cells stained with propidium iodide (IP), YO-PRO1 and both fluorochromes (merge) at 6 h, 12 h and 24 h of treatment with respect to the total cell number quantified in brightfield. Data are expressed as the mean ± SEM of four independent fields. The ANOVA test was used for statistical analysis, followed by Tukey’s post hoc test. * *p* < 0.05; ** *p* < 0.01; *** *p* < 0.001; **** *p* < 0.0001.

To verify the ability of SA to inhibit necrosis and P2X7 activation, mRPE cells were treated with 10 μM and 15 μM 7KCh and simultaneously stained with propidium iodide and YO-PRO1 (Figure 7A). The total number of cells labeled with each one of the fluorochromes was counted, again, at 6 h, 12 h and 24 h with respect to the total number of cells observed in brightfield (Figure 7B). The percentages of cells positive for propidium iodide and YO-PRO1 with 7KCh treatment were similar to those observed in Figure 6. The addition of 10 μM SA significantly decreased the entry of propidium iodide in evaluated mRPE cells at all times, as well as the internalization of YO-PRO1 after 12 h (Figure 7B). These data show that SA can attenuate both 7KCh-induced necrosis and P2X7 activation in mRPE cells due to the decreased internalization of propidium iodide and YO-PRO1, respectively.

### 3.5. 7KCh-Induced p38 Phosphorylation Is Mediated by P2X7 Activation

**Figure 8:**
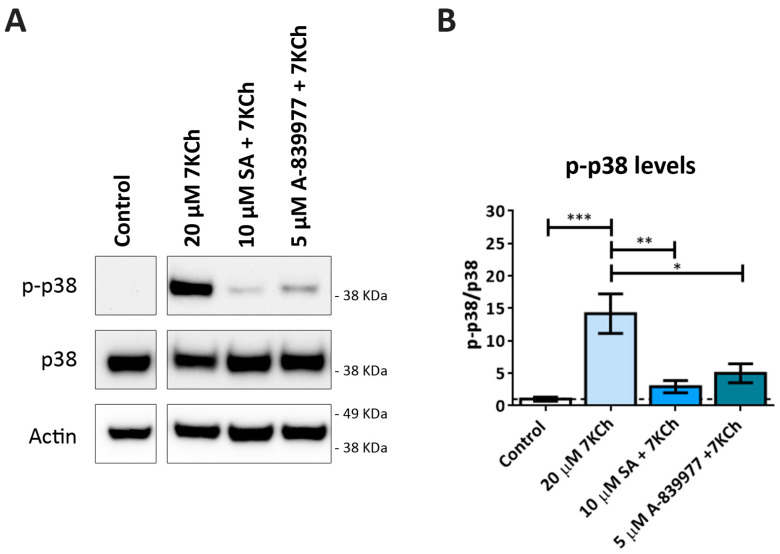
Effect of P2X7 inhibition on 7KCh-induced p38 phosphorylation. (**A**) Western blot estimation of phosphorylated p38 (p-p38) levels in mRPE cells exposed to 20 μM 7KCh alone or in combination with 10 μM SA or 5 μM A-839977 for 24 h. (**B**) Quantification of p-p38 levels over p38 levels. Data are represented as mean ± SEM of three different experiments. The dashed line is a guidance mark of the control value. The ANOVA test was used for statistical analysis, followed by Tukey’s post hoc test. * *p* < 0.05; ** *p* < 0.01; *** *p* < 0.001.

As in our previous work, the participation of p38 in the induction of cell death in response to 7KCh in mRPE cells was observed [34]. Therefore, we decided to assess the effect of P2X7 inhibition on p38 activation. The p38 phosphorylation status was evaluated by Western blot in mRPE cells treated with 20 μM 7KCh in combination with 10 μM SA and 5 μM A-839977 for 24 h (Figure 8A). A significant decrease in p38 phosphorylation levels was observed with the addition of both SA and A-839977 compared to 7KCh alone (Figure 8B). Although the effect of P2X7 inhibition on p38 activation was less than with SA treatment, these results indicate that at least an important part of p38 phosphorylation is mediated by the activation of P2X7.

### 3.6. 7KCh Treatment Does Not Induce Classical Pyroptosis Mediated by GSDMD and NLRP3 in mRPE Cells

**Figure 9:**
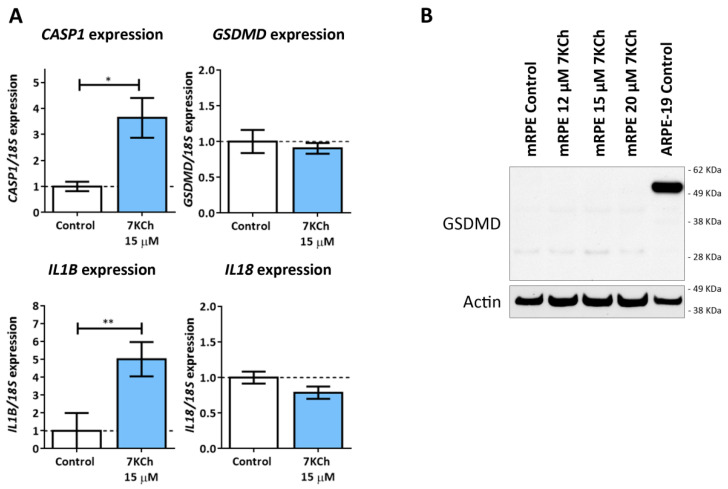
Classic pyroptosis in the cytotoxic response induced by 7KCh in mRPE cells. (**A**) Quantification of RT-qPCR of *CASP1*, *GSDMD*, *IL1B* and *IL18* expression levels in mRPE cells treated with 15 μM 7KCh for 24 h, normalized with respect to *18S* expression. (**B**) Western blot detection of GSDMD in mRPE cells exposed to 12–20 μM 7KCh. Statistical analysis in (**A**) was carried out using Student’s *t*-test. Data are expressed over the control and represented as mean ± SEM of at least three different experiments. The dashed line is a guidance mark of the control value. * *p* < 0.05; ** *p* < 0.01.

The last type of cell death to be evaluated was pyroptosis, with which the activation of P2X7 has been also associated [44]. To validate the involvement of this signaling pathway, the expression levels of *CASP1*, *NLRP3*, *GSDMD*, *IL1B* and *IL18* were evaluated in mRPE cells treated with 15 μM 7KCh by RT-qPCR (Figure 9A). An increase in the expression levels of *CASP1* and *IL1B* was observed. No change was detected in *GSDMD* or *IL18* expression levels, and, in the case of *NLRP3*, its expression could not even be quantified. On the other hand, the inhibition of Caspase 1 was not able to attenuate either the inflammation or the toxicity induced by 15 μM 7KCh in mRPE cells. Furthermore, we could not detect GSDMD activation by Western blot (Figure 9B) in mRPE cells treated with 7KCh. In fact, the band corresponding to the complete protein of around 53 KDa could not be detected in any of the mRPE cell samples. This band could be observed in the human RPE cell line ARPE-19, used as the positive control. Instead, two smaller bands, possibly non-specific, were observed in the mRPE cell samples. Taken together, these results suggest that classical pyroptosis is not activated in the 7KCh-induced cytotoxic response in mRPE cells.

### 3.7. 7KCh Induces an Alternative Pyroptosis Mechanism Mediated by the Activation of P2X7, p38 and GSDME

**Figure 10:**
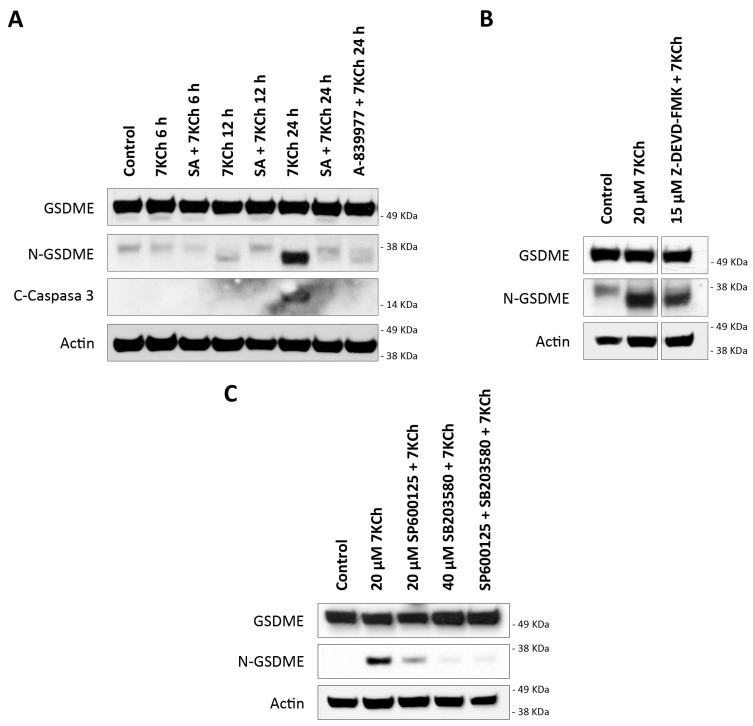
GSDME activation in mRPE cells exposed to 7KCh. (**A**) Western blot detection of GSDME cleavage (N-GSDME) and Caspase 3 cleavage (C-Caspase 3) in mRPE cells treated with 20 μM 7KCh alone or in combination with 10 μM SA at 6–24 h, and in combination with 5 μM A-839977 at 24 h. (**B**) Western blot detection of N-GSDME in mRPE cells treated with 20 μM 7KCh alone or in combination with 15 μM Z-DEVD-FMK for 24 h. (**C**) Western blot detection of N-GSDME in mRPE cells treated with 20 μM 7KCh alone or with 20 μM SP600125 and/or 40 μM SB203580 for 24 h. SP600125 and SB203580 were added with pretreatment for 2 h with respect to 7KCh.

The data obtained in our previous work from the transcriptome of mRPE cells showed an increase in the expression levels of the DFNA5 gene in cells treated with 15 μM 7KCh [34]. This gene codes for the GSDME protein, belonging to the same family of proteins as GSDMD and with a similar function, but with a difference in that it is activated by Caspase 3 [45]. The ability of 7KCh to promote the processing and activation of GSDME (N-GSDME) and Caspase 3 (C-Caspase 3) was verified by Western blot in mRPE cells treated with 20 μM alone or in combination with 10 μM SA for 6 h, 12 h and 24 h, or with the P2X7 inhibitor A-839977 at a concentration of 5 μM after 24 h of treatment (Figure 10A). The band corresponding to the active form of GSDME, of approximately 35 KDa, was observed after 12 h of treatment, with 20 μM 7KCh, reaching its highest levels of activation at 24 h. Caspase 3 activation was not detected until 24 h of exposition to 7KCh and, in addition, a long exposure of about 17–19 KDa was necessary to be able to visualize this band, showing a minimal activation of Caspase 3 despite the evident activation of GSMDE. Moreover, Caspase 3 inhibition could not prevent GSDME activation (Figure 10B), suggesting that there must be an alternative protease to Caspase 3 capable of promoting the cleavage of the N-terminus of GSDME. The addition of 10 μM SA reduced the activation of GSDME both at 12 h and 24 h, as well as Caspase 3 cleavage at 24 h. Treatment with 5 μM A-839977 could also prevent GSMDE and Caspase 3 activation at 24 h, validating the role of P2X7 in 7KCh-induced GSDME-mediated pyroptosis in mRPE cells.

On the other hand, the transcription of *DFNA5* is promoted by p53 [45]. Among the kinases capable of activating p53 by phosphorylation are both JNK and p38, previously described by our research group as being activated in response to 7KCh in mRPE cells [34]. The effect of JNK inhibition with 20 μM SP600125 and p38 inhibition with 40 μM SB203580 on GSMDE activation induced by 20 μM 7KCh was evaluated by Western blot (Figure 10C). Only treatment with SB203580, or with a combination of SP600125 with SB203580, could prevent GSDME activation. With SP600125 treatment, however, GSDME activation was still observed, albeit slightly less than with 7KCh treatment alone. This suggests that the p38-mediated phosphorylation of p53 could be responsible for the increased levels of *DFNA5* transcription and, taken together, these results show an alternative pyroptotic mechanism in response to 7KCh in mRPE cells mediated by the activation of P2X7, p38 and GSDME.

## 4. Discussion

In this work, we described a new mechanism of cell death induced by 7KCh in mRPE cells mediated by necrosis and alternative pyroptosis associated with GSDME. Once again, SA treatment was able to successfully attenuate this signaling, highlighting the importance of SA as a functional antagonist of 7KCh.

First of all, we evaluated the role of apoptosis in the cytotoxic response of 7KCh. Apoptosis and oxyapoptophagy are the main cell death mechanisms described for 7KCh, and, in general, for oxysterols [25,29,46,47,48,49,50,51,52,53,54,55,56]. Oxyapoptophagy is a type of cell death characteristic of oxysterols that combines the activation of apoptosis and autophagy [25,29,49,50,51,52,54,56]. In our previous work, we showed alterations in the expression of several genes associated with apoptosis with 7KCh-treatment in mRPE cells, such as *APAF1*, *GADD45A*, *PMAIP1* and *TP53I3* [34], suggesting that apoptosis is induced in response to 7KCh in these cells. However, the data obtained in the present work did not support the participation of apoptosis, and consequently, oxyapoptophagy in the cytotoxic response of 7KCh in retinal cells. To begin with, Caspase 3 inhibition with Z-DEVD-FMK could not attenuate 7KCh-induced cell death in mRPE cells (Figure 3B) and, despite the cleavage of Caspase 3 observed by Western blot (Figure 10A), long exposures of the films (more than 1 h of exposition) were needed in order to visualize the band, which also appeared when cell death had been already triggered (Figure 10A). This suggests that, although Caspase 3 is cleavage, its activation is not determinative for the induction of cell death in response to 7KCh. Moreover, when protein synthesis activation, which is needed for apoptosis activation [41,42], was inhibited with CHX, no change in cell viability was observed either (Figure 3B), nor could the DNA laddering fragmentation pattern characteristic of apoptosis be detected (Figure 3C). Therefore, although there might be some induction of apoptosis in response to 7KCh, its activation is late and not decisive for triggering cell death induced by this oxysterol in mRPE cells.

Given the absence of apoptosis, we next studied the role of necrosis in the response to 7KCh in retinal cells. Unlike apoptosis, there have not been many studies that describe an induction of necrosis in response to 7KCh [22,48,57], and, in some cases, it has been ruled out as a mechanism of cell death [51]. Treatment with 7KCh in mRPE cells increased the internalization and the number of nuclei stained with propidium iodide, which showed a necrotic morphology (Figure 4A). In addition, the percentage of nuclei stained with propidium iodide with 15 μM 7KCh treatment was approximately 50% (Figure 4B), coinciding with the percentage of cell death observed in mRPE cells by MTS assay with the same dose of 7KCh (Figure 2). Moreover, treatment with Nec-1 and NSA, two necroptosis inhibitors, could attenuate 7KCh-induced toxicity in mRPE cells (Figure 4C,D). This indicates that at least an important part of the cytotoxicity caused by 7KCh in mRPE cells is mediated by necrotic processes. On the other hand, this necrosis seems to be associated with P2X7 purinoceptor activation, since its inhibition with A-839977 protected mRPE cells from 7KCh-induced toxicity (Figure 5A) and significantly decreased the internalization of propidium iodide (Figure 6). P2X7-like purinoceptors are ATP-activated receptors that act as ion channels and form pores in the membrane, allowing the entry of several molecules such as the fluorescent probe YO-PRO1, which can be used to study the aperture of these receptors [43]. We observed, with exposure to 7KCh, an increase in the number of positive nuclei for YO-PRO1 after 6 h of treatment, and practically all of the labeling with propidium iodide colocalized with YO-PRO1 staining (Figure 6 and Figure 7), validating P2X7 implication in necrosis activation induced by 7KCh in mRPE cells. SA treatment successfully reduced the internalization of both fluorochromes, implying an inhibition of both necrosis induction and P2X7 opening (Figure 7), and highlighting the therapeutic potential of this molecule.

P2X7 receptors are associated with the activation of inflammation through inflammasome formation and with various types of cell death, including apoptosis, necrosis and pyroptosis [22,43,44,58,59]. P2X7 involvement in 7KCh-induced necrosis has been previously described in ARPE-19 cells, which was independent of pannexin 1 activation [22], supporting our results. We also checked the effect of P2X7 inhibition on p38 activation, as we had previously described an implication of this protein in the induction of cell death promoted by 7KCh in mRPE cells [34]. A-839977 significantly reduced p38 phosphorylation levels induced in response to 7KCh (Figure 8), as well as SA treatment, in agreement with our previous work [34]. This indicates that an important part of p38 activation is dependent on the induction of P2X7 receptors, consistent with previous reports [43,60,61].

The last type of cell death to be evaluated was pyroptosis, since P2X7 activation has also been related to these mechanisms and pyroptotic cells are also labeled with propidium iodide [43,44,59]. Pyroptosis is a mechanism of inflammatory cell death and the canonical or classical pathway is characterized by the activation of NLRP3 inflammasome, which is responsible for the activation of Caspase 1. Once activated, Caspase 1 promotes the cleavage of the GSDMD N-terminus end, which forms pores in the membrane through which IL-1β and IL-18 are released, followed by the entire cytosolic content [42,62,63]. Although the induction of the NLRP3 inflammasome in response to 7KCh has been reported several times [18,19,24], no direct association between 7KCh and pyroptosis has been established so far. In the genome-wide analysis study carried out in our previous work, we described an increase in the gene expression of *CASP1* in 7KCh-treated cells [34]. In the present work, we confirmed this gene expression alteration by qRT-PCR and observed a modulation of *IL1B* expression in response to 7KCh (Figure 9A). However, no changes in the expression levels of *GSDMD* and *IL18* were seen, and the expression of *NLRP3* could not even be detected. Interestingly, despite detecting the expression of *GSDMD*, the band corresponding to the protein was not observed by Western blot in mRPE cells (Figure 9B). In the transcriptome analysis of our previous work, the expression of *GSDMD* could also be detected in all of the samples tested, but with a relatively low number of counts compared to the number of counts quantified in ARPE-19 cells (approximately 80 counts in ARPE-19 cells and 25 counts in mRPE cells). This could explain why, in mRPE cells, the GSDMD protein is not observed, since the levels of GSDMD would be too low to be detected, but can be observed in ARPE-19 cells, used as the positive control. Taken together, these results indicate that the canonical pyroptosis pathway is not involved in the response to 7KCh.

Transcriptome sequencing data also showed a positive regulation of *DFNA5*, the gene that codes GSDME, with 7KCh [34]. GSDME belongs to the same family of proteins as GSDMD and also acts as an effector protein for pyroptosis. The cleavage of the N-terminus end of GSDME in a ~35 KDa fragment is induced by the action of Caspase 3 instead of Caspase 1, which means that GSDME can be activated independently of the NLRP3 inflammasome [45,62,63,64,65,66]. Both GSDME and Caspase 3 cleavage were observed in mRPE cells treated with 20 μM 7KCh (Figure 10A). However, as mentioned above, long exposures were required for the detection of Caspase 3, whereas this was not the case for the visualization of active GSDME. Furthermore, contrary to what might be expected, it was observed that while GSDME activation was already detected after 12 h of exposure to 20 μM 7KCh, Caspase 3 cleavage was not visualized until 24 h. Similarly, Caspase 3 inhibition could not prevent GSDME cleavage (Figure 10B), suggesting that an alternative protein to Caspase 3 must exist, being capable of promoting GSDME activation. In this way, it seems to be GSDME itself that increases Caspase 3 activation in a positive feedback loop, since it has been described that GSDME can also promote Caspase 3 induction [45,65].

Regarding the cleavage of GSDME, the appearance, on some occasions, of two intermediate bands in mRPE cells is striking (Figure 10A): a lower band than that corresponding to the complete protein, and another of about 38 KDa higher than that corresponding to active GSDME (~35 KDa). These intermediate bands can be even seen in control cells, suggesting some basal GSDME processing under normal conditions, but without the protein becoming activated. In those treatments where there is no cell and GSDME has not been activated yet, such as the control or treatment with 20 μM 7KCh at 6 h, these intermediate bands can be seen. When GSDME begins to activate, as in 20 μM 7KCh after 12 h of treatment, the upper intermediate band disappears, although the ~38 KDa band can still be seen. In contrast, when GSDME is fully activated, such as during 24 h of treatment with 20 μM 7KCh, the ~35 KDa band is most strongly detected, while the two intermediate bands have disappeared, indicating that GSDME is fully cleaved and active. The presence of several intermediate bands of GSDME has also been visualized in several previous studies [67,68,69], although, for the moment, there has been no work that confirms that they are intermediate cleavages.

*DFNA5* is associated with the development of sensorineural deafness and, in tumor cells, it has been shown to act as a tumor suppressor gene. In different tissues and cell types, it has been described that *DFNA5* transcription is induced by p53 through a binding site for this protein located in intron 1 of the gene [45,70,71]. The regulation and activation of p53 is complex, since it presents several phosphorylation, acetylation, methylation and ubiquitination sites, and it is a target of multiple enzymes. Among proteins capable of phosphorylate p53, we can find both JNK and p38 [45,72]. The inhibition of p38, but not JNK, attenuated GSDME activation in mRPE cells (Figure 10C), indirectly suggesting that the activation of p53 through p38 might be responsible for the increased expression of the gene that codes for GSDME. These results are in agreement with our previous work, where we showed that p38, but not JNK, was implicated in cell death induced by 7KCh in mRPE cells [34]. P2X7 inhibition not only decreased p38 phosphorylation, as mentioned above (Figure 8), but also reduced GSDME activation (Figure 10A). This confirms the involvement of P2X7 in pyroptosis and suggests that 7KCh activates an alternative pyroptotic mechanism mediated by P2X7, p38 and GSDME in mRPE cells not previously described. On the other hand, treatment with 10 μM SA, in addition to decreasing the activation of both P2X7 (Figure 7) and p38 (Figure 6), was able to prevent GSDME cleavage at 12 h and 24 h of treatment, as well as Caspase 3 cleavage at 24 h (Figure 10A). These results indicate that SA acts at various levels of the 7KCh-induced response and, once again, highlights its therapeutic potential, postulating it as the most potent functional antagonist of 7KCh in retinal cells.

GSDME activation could explain why, although there is a little induction, apoptosis is not fully triggered, since when Caspase 3 activates GSDME, the initial apoptotic response culminates in pyroptosis instead of apoptosis [45]. In addition, pyroptosis is sometimes considered a secondary necrosis or one of the types of regulated necrosis, and some studies have described that GSDME can induce necrosis [45,66,67,73,74,75]. Thus, the necrosis and pyroptosis observed in mRPE cells in response to 7KCh might be related. The activation of P2X7 would first induce the pyroptotic response, which would end up triggering necrosis. This could explain why, with 7KCh-treatment, the internalization of YO-PRO1 is observed before propidium iodide labeling, and there is always a percentage of cells only stained with YO-PRO1 that, as cell death progresses, are also stained with propidium iodide (Figure 6 and Figure 7).

7KCh is one of the main molecules accumulated in drusen and its relationship with the development of AMD is well established [17,18,19,20,22,23,24,30,34,35,76]. In the current work, we showed that 7KCh induces a cell death mechanism in retinal cells characterized by the induction of necrosis and pyroptosis, and mediated by the activation of P2X7, p38 and GSDME. There have been several studies that have associated P2X7 activation with the pathogenesis of AMD, both in vivo and in vitro [22,44,59]. Recent publications have also described the induction of GSDME in retinal cells exposed to all-trans-retinal, another molecule associated with the development of AMD [65,77]. Thus, the characterization of the response induced by 7KCh in retinal cells offers new opportunities to search for alternative therapeutic targets for this disease. On the other hand, we have demonstrated, once again, the ability of SA to counteract the harmful effect of 7KCh on mRPE cells, in agreement with previous studies [23,24,34,35,36], being able to act at various levels of the response induced by this oxysterol. For these reasons, SA is currently the molecule that works best to attenuate the effect of 7KCh in retinal cells and it is presented as a new opportunity to complement or improve the therapies used to treat AMD. Although there is still a long way to go and further research is needed, the present work is a step forward in the study of the mechanism of action of 7KCh in the pathogenesis of AMD and in the therapeutic potential of SA to target the effect of this oxysterol.

## Figures and Tables

**Table 1:** Primers used in this work in mRPE cells.

Gene	Oligonucleotide Sequence
*18S*—Forward	5′-ATGCTCTTAGCTGAGTGTCCCG-3′
*18S*—Reverse	5′-ATTCCTAGCTGCGGTATCCAGG-3′
*CASP1*—Forward	5′-CCATTCCCCTCCTACCCTGA-3′
*CASP1*—Reverse	5′-TGCTTCGTCTTCCTTTTTGGC-3′
*GSDMD*—Forward	5′-CGAAGATCACGGGTGGGG-3′
*GSDMD*—Reverse	5′-GCAGGATTTTGTGCTCTGGC-3′
*IL1B*—Forward	5′-ACACTCCGGGATGCACAGC-3′
*IL1B*—Reverse	5′-CCCAAGGCCACAGGTATTTT-3′
*IL18*—Forward	5′-ACCAAGGAAATCGGCCCCTA-3′
*IL18*—Reverse	5′-CCATACCTCTAGGCTGGCTATC-3′
*NLRP3*—Forward	5′-GGCTGGAGCTGTTGAAATGG-3′
*NLRP3*—Reverse	5′-CCCTTTGCACGAAGTCCTCC-3′

## Data Availability

Not applicable.

## Abbreviations

7KCh	7-Ketocholesterol
AMD	age-related macular degeneration
CBX	carbenoxolone
CHX	cicloheximide
CNV	choroidal neovascularization
mRPE	monkey retinal pigment epithelium cells
Nec-1	necrostatine-1
NSA	necrosulfonamide
PVDF	polyvinylidene difluoride
qRT-PCR	quantitative real-time PCR
RPE	retinal pigment epithelium
SA	sterculic acid
SCD1	stearoyl coenzyme-A desaturase 1
SDS	sodium dodecyl sulphate
SEM	standard error of the means
TBS	tris buffered saline
UPR	unfolded protein response

## References

[1] Deng Y., Qiao L., Du M., Qu C., Wan L., Li J., Huang L. (2022). Age-related macular degeneration: Epidemiology, genetics, pathophysiology, diagnosis, and targeted therapy. Genes Dis..

[2] Mehrzadi S., Hemati K., Reiter R.J., Hosseinzadeh A. (2020). Mitochondrial dysfunction in age-related macular degeneration: Melatonin as a potential treatment. Expert Opin. Ther. Targets.

[3] Taylor A. (2012). Introduction to the issue regarding research regarding age related macular degeneration. Mol. Asp. Med..

[4] Xu Q., Cao S., Rajapakse S., Matsubara J.A. (2018). Understanding AMD by analogy: Systematic review of lipid-related common pathogenic mechanisms in AMD, AD, AS and GN. Lipids Health Dis..

[5] Stahl A. (2020). The Diagnosis and Treatment of Age-Related Macular Degeneration. Dtsch. Arztebl. Int..

[6] Thomas C.J., Mirza R.G., Gill M.K. (2021). Age-Related Macular Degeneration. Med. Clin. N. Am..

[7] Curcio C.A. (2018). Antecedents of Soft Drusen, the Specific Deposits of Age-Related Macular Degeneration, in the Biology of Human Macula. Investig. Ophthalmol. Vis. Sci..

[8] Curcio C.A. (2018). Soft Drusen in Age-Related Macular Degeneration: Biology and Targeting Via the Oil Spill Strategies. Investig. Ophthalmol. Vis. Sci..

[9] Spaide R.F., Ooto S., Curcio C.A. (2018). Subretinal drusenoid deposits AKA pseudodrusen. Surv. Ophthalmol..

[10] Curcio C.A., Johnson M., Rudolf M., Huang J.D. (2011). The oil spill in ageing Bruch membrane. Br. J. Ophthalmol..

[11] Abokyi S., To C.H., Lam T.T., Tse D.Y. (2020). Central Role of Oxidative Stress in Age-Related Macular Degeneration: Evidence from a Review of the Molecular Mechanisms and Animal Models. Oxidative Med. Cell. Longev..

[12] Garcia-Garcia J., Usategui-Martin R., Sanabria M.R., Fernandez-Perez E., Telleria J.J., Coco-Martin R.M. (2022). Pathophysiology of Age-Related Macular Degeneration. Implications for Treatment. Ophthalmic Res..

[13] Moreira E.F., Larrayoz I.M., Lee J.W., Rodriguez I.R. (2009). 7-Ketocholesterol is present in lipid deposits in the primate retina: Potential implication in the induction of VEGF and CNV formation. Investig. Ophthalmol. Vis. Sci..

[14] Bobadilla M., Pariente A., Oca A.I., Pelaez R., Perez-Sala A., Larrayoz I.M. (2022). Biomarkers as Predictive Factors of Anti-VEGF Response. Biomedicines.

[15] Oca A.I., Perez-Sala A., Pariente A., Ochoa R., Velilla S., Pelaez R., Larrayoz I.M. (2021). Predictive Biomarkers of Age-Related Macular Degeneration Response to Anti-VEGF Treatment. J. Pers. Med..

[16] Mettu P.S., Allingham M.J., Cousins S.W. (2021). Incomplete response to Anti-VEGF therapy in neovascular AMD: Exploring disease mechanisms and therapeutic opportunities. Prog. Retin. Eye Res..

[17] Poli G., Biasi F., Leonarduzzi G. (2013). Oxysterols in the pathogenesis of major chronic diseases. Redox Biol..

[18] Indaram M., Ma W., Zhao L., Fariss R.N., Rodriguez I.R., Wong W.T. (2015). 7-Ketocholesterol increases retinal microglial migration, activation, and angiogenicity: A potential pathogenic mechanism underlying age-related macular degeneration. Sci. Rep..

[19] Shi G., Chen S., Wandu W.S., Ogbeifun O., Nugent L.F., Maminishkis A., Hinshaw S.J., Rodriguez I.R., Gery I. (2015). Inflammasomes Induced by 7-Ketocholesterol and Other Stimuli in RPE and in Bone Marrow-Derived Cells Differ Markedly in Their Production of IL-1beta and IL-18. Investig. Ophthalmol. Vis. Sci..

[20] Amaral J., Lee J.W., Chou J., Campos M.M., Rodriguez I.R. (2013). 7-Ketocholesterol induces inflammation and angiogenesis in vivo: A novel rat model. PLoS ONE.

[21] Rodriguez I.R., Clark M.E., Lee J.W., Curcio C.A. (2014). 7-ketocholesterol accumulates in ocular tissues as a consequence of aging and is present in high levels in drusen. Exp. Eye Res..

[22] Olivier E., Dutot M., Regazzetti A., Leguillier T., Dargere D., Auzeil N., Laprevote O., Rat P. (2016). P2X7-pannexin-1 and amyloid beta-induced oxysterol input in human retinal cell: Role in age-related macular degeneration?. Biochimie.

[23] Huang J.D., Amaral J., Lee J.W., Larrayoz I.M., Rodriguez I.R. (2012). Sterculic acid antagonizes 7-ketocholesterol-mediated inflammation and inhibits choroidal neovascularization. Biochim. Biophys. Acta.

[24] Huang J.D., Amaral J., Lee J.W., Rodriguez I.R. (2014). 7-Ketocholesterol-induced inflammation signals mostly through the TLR4 receptor both in vitro and in vivo. PLoS ONE.

[25] Leoni V., Nury T., Vejux A., Zarrouk A., Caccia C., Debbabi M., Fromont A., Sghaier R., Moreau T., Lizard G. (2017). Mitochondrial dysfunctions in 7-ketocholesterol-treated 158N oligodendrocytes without or with alpha-tocopherol: Impacts on the cellular profil of tricarboxylic cycle-associated organic acids, long chain saturated and unsaturated fatty acids, oxysterols, cholesterol and cholesterol precursors. J. Steroid Biochem. Mol. Biol..

[26] Sul O.J., Li G., Kim J.E., Kim E.S., Choi H.S. (2021). 7-ketocholesterol enhances autophagy via the ROS-TFEB signaling pathway in osteoclasts. J. Nutr. Biochem..

[27] Tani M., Kamata Y., Deushi M., Osaka M., Yoshida M. (2018). 7-Ketocholesterol enhances leukocyte adhesion to endothelial cells via p38MAPK pathway. PLoS ONE.

[28] Yang C., Xie L., Gu Q., Qiu Q., Wu X., Yin L. (2019). 7-Ketocholesterol disturbs RPE cells phagocytosis of the outer segment of photoreceptor and induces inflammation through ERK signaling pathway. Exp. Eye Res..

[29] Nury T., Sghaier R., Zarrouk A., Menetrier F., Uzun T., Leoni V., Caccia C., Meddeb W., Namsi A., Sassi K. (2018). Induction of peroxisomal changes in oligodendrocytes treated with 7-ketocholesterol: Attenuation by alpha-tocopherol. Biochimie.

[30] Wang H., Ramshekar A., Kunz E., Hartnett M.E. (2021). 7-ketocholesterol induces endothelial-mesenchymal transition and promotes fibrosis: Implications in neovascular age-related macular degeneration and treatment. Angiogenesis.

[31] Vejux A., Abed-Vieillard D., Hajji K., Zarrouk A., Mackrill J.J., Ghosh S., Nury T., Yammine A., Zaibi M., Mihoubi W. (2020). 7-Ketocholesterol and 7beta-hydroxycholesterol: In vitro and animal models used to characterize their activities and to identify molecules preventing their toxicity. Biochem. Pharmacol..

[32] Brahmi F., Vejux A., Sghaier R., Zarrouk A., Nury T., Meddeb W., Rezig L., Namsi A., Sassi K., Yammine A. (2019). Prevention of 7-ketocholesterol-induced side effects by natural compounds. Crit. Rev. Food Sci. Nutr..

[33] Pariente A., Pelaez R., Perez-Sala A., Larrayoz I.M. (2019). Inflammatory and cell death mechanisms induced by 7-ketocholesterol in the retina. Implications for age-related macular degeneration. Exp. Eye Res..

[34] Pariente A., Perez-Sala A., Ochoa R., Bobadilla M., Villanueva-Martinez A., Pelaez R., Larrayoz I.M. (2023). Identification of 7-Ketocholesterol-Modulated Pathways and Sterculic Acid Protective Effect in Retinal Pigmented Epithelium Cells by Using Genome-Wide Transcriptomic Analysis. Int. J. Mol. Sci..

[35] Pariente A., Perez-Sala A., Ochoa R., Pelaez R., Larrayoz I.M. (2020). Genome-Wide Transcriptomic Analysis Identifies Pathways Regulated by Sterculic Acid in Retinal Pigmented Epithelium Cells. Cells.

[36] William S., Duncan T., Redmond T.M. (2022). Pretreatment of human retinal pigment epithelial cells with sterculic acid forestalls fenretinide-induced apoptosis. Sci. Rep..

[37] Herrera-Meza M.S., Mendoza-Lopez M.R., Garcia-Barradas O., Sanchez-Otero M.G., Silva-Hernandez E.R., Angulo J.O., Oliart-Ros R.M. (2013). Dietary anhydrous milk fat naturally enriched with conjugated linoleic acid and vaccenic acid modify cardiovascular risk biomarkers in spontaneously hypertensive rats. Int. J. Food Sci. Nutr..

[38] Pelaez R., Pariente A., Perez-Sala A., Larrayoz I.M. (2020). Sterculic Acid: The Mechanisms of Action beyond Stearoyl-CoA Desaturase Inhibition and Therapeutic Opportunities in Human Diseases. Cells.

[39] Ortinau L.C., Nickelson K.J., Stromsdorfer K.L., Naik C.Y., Pickering R.T., Haynes R.A., Fritsche K.L., Perfield J.W. (2013). Sterculic oil, a natural inhibitor of SCD1, improves the metabolic state of obese OLETF rats. Obesity.

[40] Becerra S.P., Fariss R.N., Wu Y.Q., Montuenga L.M., Wong P., Pfeffer B.A. (2004). Pigment epithelium-derived factor in the monkey retinal pigment epithelium and interphotoreceptor matrix: Apical secretion and distribution. Exp. Eye Res..

[41] Nikoletopoulou V., Markaki M., Palikaras K., Tavernarakis N. (2013). Crosstalk between apoptosis, necrosis and autophagy. Biochim. Biophys. Acta.

[42] Tang D., Kang R., Berghe T.V., Vandenabeele P., Kroemer G. (2019). The molecular machinery of regulated cell death. Cell Res..

[43] Kopp R., Krautloher A., Ramirez-Fernandez A., Nicke A. (2019). P2X7 Interactions and Signaling—Making Head or Tail of It. Front. Mol. Neurosci..

[44] Kerur N., Hirano Y., Tarallo V., Fowler B.J., Bastos-Carvalho A., Yasuma T., Yasuma R., Kim Y., Hinton D.R., Kirschning C.J. (2013). TLR-independent and P2X7-dependent signaling mediate Alu RNA-induced NLRP3 inflammasome activation in geographic atrophy. Investig. Ophthalmol. Vis. Sci..

[45] Jiang M., Qi L., Li L., Li Y. (2020). The caspase-3/GSDME signal pathway as a switch between apoptosis and pyroptosis in cancer. Cell Death Discov..

[46] Chang M.C., Chen Y.J., Liou E.J., Tseng W.Y., Chan C.P., Lin H.J., Liao W.C., Chang Y.C., Jeng P.Y., Jeng J.H. (2016). 7-Ketocholesterol induces ATM/ATR, Chk1/Chk2, PI3K/Akt signalings, cytotoxicity and IL-8 production in endothelial cells. Oncotarget.

[47] Kim A., Nam Y.J., Lee C.S. (2017). Taxifolin reduces the cholesterol oxidation product-induced neuronal apoptosis by suppressing the Akt and NF-kappaB activation-mediated cell death. Brain Res. Bull..

[48] Koh S.S., Ooi S.C., Lui N.M., Qiong C., Ho L.T., Cheah I.K., Halliwell B., Herr D.R., Ong W.Y. (2021). Effect of Ergothioneine on 7-Ketocholesterol-Induced Endothelial Injury. Neuromolecular Med..

[49] Nury T., Zarrouk A., Mackrill J.J., Samadi M., Durand P., Riedinger J.M., Doria M., Vejux A., Limagne E., Delmas D. (2015). Induction of oxiapoptophagy on 158N murine oligodendrocytes treated by 7-ketocholesterol-, 7beta-hydroxycholesterol-, or 24(S)-hydroxycholesterol: Protective effects of alpha-tocopherol and docosahexaenoic acid (DHA; C22:6 n-3). Steroids.

[50] Nury T., Zarrouk A., Vejux A., Doria M., Riedinger J.M., Delage-Mourroux R., Lizard G. (2014). Induction of oxiapoptophagy, a mixed mode of cell death associated with oxidative stress, apoptosis and autophagy, on 7-ketocholesterol-treated 158N murine oligodendrocytes: Impairment by alpha-tocopherol. Biochem. Biophys. Res. Commun..

[51] Paz J.L., Levy D., Oliveira B.A., de Melo T.C., de Freitas F.A., Reichert C.O., Rodrigues A., Pereira J., Bydlowski S.P. (2019). 7-Ketocholesterol Promotes Oxiapoptophagy in Bone Marrow Mesenchymal Stem Cell from Patients with Acute Myeloid Leukemia. Cells.

[52] Ragot K., Delmas D., Athias A., Nury T., Baarine M., Lizard G. (2011). alpha-Tocopherol impairs 7-ketocholesterol-induced caspase-3-dependent apoptosis involving GSK-3 activation and Mcl-1 degradation on 158N murine oligodendrocytes. Chem. Phys. Lipids.

[53] Ragot K., Mackrill J.J., Zarrouk A., Nury T., Aires V., Jacquin A., Athias A., Pais de Barros J.P., Vejux A., Riedinger J.M. (2013). Absence of correlation between oxysterol accumulation in lipid raft microdomains, calcium increase, and apoptosis induction on 158N murine oligodendrocytes. Biochem. Pharmacol..

[54] Soh S., Ong W.Y. (2022). Effect of Withanolide A on 7-Ketocholesterol Induced Cytotoxicity in hCMEC/D3 Brain Endothelial Cells. Cells.

[55] Xiao Q., Che X., Cai B., Tao Z., Zhang H., Shao Q., Pu J. (2020). Macrophage autophagy regulates mitochondria-mediated apoptosis and inhibits necrotic core formation in vulnerable plaques. J. Cell. Mol. Med..

[56] Yammine A., Zarrouk A., Nury T., Vejux A., Latruffe N., Vervandier-Fasseur D., Samadi M., Mackrill J.J., Greige-Gerges H., Auezova L. (2020). Prevention by Dietary Polyphenols (Resveratrol, Quercetin, Apigenin) Against 7-Ketocholesterol-Induced Oxiapoptophagy in Neuronal N2a Cells: Potential Interest for the Treatment of Neurodegenerative and Age-Related Diseases. Cells.

[57] Ghelli A., Porcelli A.M., Zanna C., Rugolo M. (2002). 7-Ketocholesterol and staurosporine induce opposite changes in intracellular pH, associated with distinct types of cell death in ECV304 cells. Arch. Biochem. Biophys..

[58] Adinolfi E., Giuliani A.L., De Marchi E., Pegoraro A., Orioli E., Di Virgilio F. (2018). The P2X7 receptor: A main player in inflammation. Biochem. Pharmacol..

[59] Yang D. (2017). Targeting the P2X7 Receptor in Age-Related Macular Degeneration. Vision.

[60] Bianco F., Perrotta C., Novellino L., Francolini M., Riganti L., Menna E., Saglietti L., Schuchman E.H., Furlan R., Clementi E. (2009). Acid sphingomyelinase activity triggers microparticle release from glial cells. EMBO J..

[61] Green J.P., Souilhol C., Xanthis I., Martinez-Campesino L., Bowden N.P., Evans P.C., Wilson H.L. (2018). Atheroprone flow activates inflammation via endothelial ATP-dependent P2X7-p38 signalling. Cardiovasc. Res..

[62] Feng S., Fox D., Man S.M. (2018). Mechanisms of Gasdermin Family Members in Inflammasome Signaling and Cell Death. J. Mol. Biol..

[63] Orning P., Lien E., Fitzgerald K.A. (2019). Gasdermins and their role in immunity and inflammation. J. Exp. Med..

[64] Li Y.Q., Peng J.J., Peng J., Luo X.J. (2019). The deafness gene GSDME: Its involvement in cell apoptosis, secondary necrosis, and cancers. Naunyn Schmiedebergs Arch. Pharmacol..

[65] Liao X.X., Dai Y.Z., Zhao Y.Z., Nie K. (2022). Gasdermin E: A Prospective Target for Therapy of Diseases. Front. Pharmacol..

[66] De Schutter E., Croes L., Ibrahim J., Pauwels P., Op de Beeck K., Vandenabeele P., Van Camp G. (2021). GSDME and its role in cancer: From behind the scenes to the front of the stage. Int. J. Cancer.

[67] Wen S., Wang Z.H., Zhang C.X., Yang Y., Fan Q.L. (2020). Caspase-3 Promotes Diabetic Kidney Disease Through Gasdermin E-Mediated Progression to Secondary Necrosis During Apoptosis. Diabetes Metab. Syndr. Obes..

[68] Zhao W., Zhang L., Zhang Y., Jiang Z., Lu H., Xie Y., Han W., He J., Shi Z., Yang H. (2023). The CDK inhibitor AT7519 inhibits human glioblastoma cell growth by inducing apoptosis, pyroptosis and cell cycle arrest. Cell Death Dis..

[69] Zhou B., Ryder C.B., Dubyak G.R., Abbott D.W. (2022). Gasdermins and pannexin-1 mediate pathways of chemotherapy-induced cell lysis in hematopoietic malignancies. Sci. Signal.

[70] Masuda Y., Futamura M., Kamino H., Nakamura Y., Kitamura N., Ohnishi S., Miyamoto Y., Ichikawa H., Ohta T., Ohki M. (2006). The potential role of DFNA5, a hearing impairment gene, in p53-mediated cellular response to DNA damage. J. Hum. Genet..

[71] Op de Beeck K., Van Camp G., Thys S., Cools N., Callebaut I., Vrijens K., Van Nassauw L., Van Tendeloo V.F., Timmermans J.P., Van Laer L. (2011). The DFNA5 gene, responsible for hearing loss and involved in cancer, encodes a novel apoptosis-inducing protein. Eur. J. Hum. Genet..

[72] Jenkins L.M., Durell S.R., Mazur S.J., Appella E. (2012). p53 N-terminal phosphorylation: A defining layer of complex regulation. Carcinogenesis.

[73] Chen K.W., Demarco B., Broz P. (2020). Beyond inflammasomes: Emerging function of gasdermins during apoptosis and NETosis. EMBO J..

[74] Mazlo A., Tang Y., Jenei V., Brauman J., Yousef H., Bacsi A., Koncz G. (2022). Resolution Potential of Necrotic Cell Death Pathways. Int. J. Mol. Sci..

[75] Tixeira R., Shi B., Parkes M.A.F., Hodge A.L., Caruso S., Hulett M.D., Baxter A.A., Phan T.K., Poon I.K.H. (2018). Gasdermin E Does Not Limit Apoptotic Cell Disassembly by Promoting Early Onset of Secondary Necrosis in Jurkat T Cells and THP-1 Monocytes. Front. Immunol..

[76] Larrayoz I.M., Huang J.D., Lee J.W., Pascual I., Rodriguez I.R. (2010). 7-ketocholesterol-induced inflammation: Involvement of multiple kinase signaling pathways via NFkappaB but independently of reactive oxygen species formation. Investig. Ophthalmol. Vis. Sci..

[77] Cai B., Liao C., He D., Chen J., Han J., Lu J., Qin K., Liang W., Wu X., Liu Z. (2022). Gasdermin E mediates photoreceptor damage by all-trans-retinal in the mouse retina. J. Biol. Chem..

